# From grass to gas: microbiome dynamics of grass biomass acidification under mesophilic and thermophilic temperatures

**DOI:** 10.1186/s13068-017-0859-0

**Published:** 2017-07-03

**Authors:** Christian Abendroth, Claudia Simeonov, Juli Peretó, Oreto Antúnez, Raquel Gavidia, Olaf Luschnig, Manuel Porcar

**Affiliations:** 1 Cavanilles Institute of Biodiversity and Evolutionary Biology, Universitat de València, C/ José Beltran 2, 46980 Paterna, Spain; 2 Institute for Integrative Systems Biology (I2SysBio, Universitat de València-CSIC), C/ José Beltran 2, 46980 Paterna, Spain; 3 Robert Boyle Institut e.V., Im Steinfeld 10, 07751 Jena, Germany; 4 Departament de Bioquímica i Biologia Molecular, Universitat de València, Paterna, Spain; 5 Servei Central de Suport a la Investigació Experimental (SCSIE), Universitat de València-CSIC, Paterna, Spain; 6 Bio H2 Energy GmbH, Im Steinfeld 10, 07751 Jena, Germany; 7 Darwin Bioprospecting Excellence, S.L. Parc Cientific Universitat de Valencia, C/ Catedrático Agustín Escardino Benlloch, 9, 46980 Paterna, Valencia Spain; 8 Institute for Integrative Systems Biology (I2SysBio, Universitat de València-CSIC), Postal Code 22085, 46071 Paterna, València Spain

## Abstract

**Background:**

Separating acidification and methanogenic steps in anaerobic digestion processes can help to optimize the process and contribute to producing valuable sub-products such as methane, hydrogen and organic acids. However, the full potential of this technology has not been fully explored yet. To assess the underlying fermentation process in more detail, a combination of high-throughput sequencing and proteomics on the acidification step of plant material (grass) at both mesophilic and thermophilic temperatures (37 and 55 °C, respectively) was applied for the first time.

**Results:**

High-strength liquor from acidified grass biomass exhibited a low biodiversity, which differed greatly depending on temperature. It was dominated by Bacteroidetes and Firmicutes at 37 °C, and by Firmicutes and Proteobacteria at 55 °C. At the methane stage, *Methanosaeta*, *Methanomicrobium* and *Methanosarcina* proved to be highly sensitive to environmental changes as their abundance in the seed sludges dropped dramatically after transferring the seed sludges from the respective reactors into the experimental setup. Further, an increase in Actinobacteria coincided with reduced biogas production at the end of the experiment. Over 1700 proteins were quantified from the first cycle of acidification samples using label-free quantitative proteome analysis and searching protein databases. The most abundant proteins included an almost complete set of glycolytic enzymes indicating that the microbial population is basically engaged in the degradation and catabolism of sugars. Differences in protein abundances clearly separated samples into two clusters corresponding to culture temperature. More differentially expressed proteins were found under mesophilic (120) than thermophilic (5) conditions.

**Conclusion:**

Our results are the first multi-omics characterisation of a two-stage biogas production system with separated acidification and suggest that screening approaches targeting specific taxa such as *Methanosaeta*, *Methanomicrobium* and *Methanosarcina* could be useful diagnostic tools as indicators of environmental changes such as temperature or oxidative stress or, as in the case of Actinobacteria, they could be used as a proxy of the gas production potential of anaerobic digesters. Metaproteome analyses only detected significant expression differences in mesophilic samples, whereas thermophilic samples showed more stable protein composition with an abundance of chaperones suggesting a role in protein stability under thermal stress.

**Electronic supplementary material:**

The online version of this article (doi:10.1186/s13068-017-0859-0) contains supplementary material, which is available to authorized users.

## Background

Anaerobic digestion is a promising technology for biofuel production, and has been the object of research for over 100 years [1, 2]. The anaerobic digestion process consists of four stages: hydrolysis, acidogenesis, acetogenesis and methanogenesis. During the first three stages, hydrogen and acetate are formed as intermediary products, which are then converted into methane and carbon dioxide during methanogenesis [3]. Countless works have been published characterizing those stages or comparing different substrates for co-digestion and reactor configurations. Furthermore, substantial efforts have been made in recent decades to shed light on the underlying microbial biocoenosis of anaerobic digestion processes. The first determinations of taxonomic profiles appeared in the 90 s [4, 5], when 16S-rDNA data from anaerobic sludges were investigated. More recently, high-throughput approaches like 16S-rDNA sequencing or metagenomics have been applied [2, 6–8], as well as proteome analyses [9, 10]. However, most of the aforementioned work focused on reactor configurations, where acidogenesis and methanogenesis occur, combined in the same reactor stage. It is well-known since the 80 s that the process can be split into multistage processes, in such a way that hydrolysis/acidogenesis occurs separately from acetogenesis/methanogenesis [11, 12]. Although it may be difficult to fully separate the underlying microbial processes (for example nitrogen-rich substrates seem to cause methanogenic contaminations in the acid-producing step [13]), improved biogas production has been reported using a separated setup. For example, in 1988 authors described a rumen-derived microbial community optimally fermenting cellulose in a separated acidification step [14]. Others report that some practices such as shock loading (high loads of substrate that cause accumulation of volatile fatty acids, VFA) increase hydrogen formation at pH < 6.5 [15]. As pH values between 4 and 6.5 are common during acidification [16–18] and methanogenesis is inhibited at either low pH or high VFA concentration [19], this renders hydrogen production in the acidification stage as a valuable sub-product in addition to the methane [20]. Additionally, a high concentration of acetic acid is known to improve chemical hydrolysis [21]. Even though hydrogen production in seed sludges with diverse microbiomes is highly unpredictable, a few previous reports have explored the possible production of hydrogen [22–24], by, for example, immobilization of hydrogen-producing bacteria [23, 24].

Separated acidification has been proposed as the best technology to produce organic acids like lactic, butyric and acetic acid, even though it is still complicated to extract organic acids from the fermentation process [25].

The benefits of separated acidification cannot be fully explored without a deeper knowledge of the underlying microbial communities. Currently, such knowledge is very fragmentary. For example, it is known that fermentation of 52.85 g/L of rice straw at 39.23 °C and pH 10.0 leads to an increase in the families Ruminococcaceae, Bacteroidaceae, Porphyromonadaceae and Lachnospiraceae [26]; or that the acidification of alginate correlates with high titres of *Bacteroides*- and *Clostridium*-related microorganisms [27]. Proteomics has been used to study standard, one-step digestion plants without separated acidification [9, 10, 28], but there are no detailed proteomics studies of a separated acidification stage to date. In order to bridge this gap and to finely characterize one of the most important phases of the biogas production process, the dynamic behaviour of grass acidification processes at mesophilic and thermophilic temperatures (37 and 55 °C, respectively) was monitored through both proteomics and 16S-rDNA analysis. The efficient use of lignocellulosic biomass as a feedstock is an active research area of high interest [30]. In the present work grass was chosen because of its potential as a renewable energy source [29].

## Results and discussion

### 16S-rDNA-based analysis on high-strength liquor from grass acidification

**Fig. 1:**
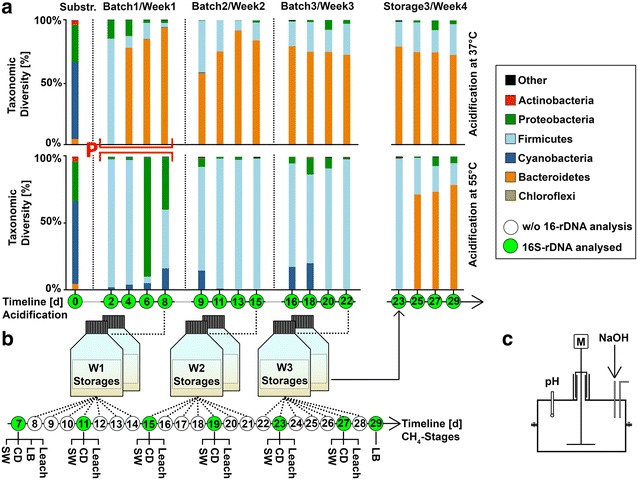
16S-rDNA-based taxonomic profiles from untreated grass substrate, samples during acidification and stored hydrolysate, at 37 °C (*upper panel*) and 55 °C (*lower panel*) (**a**). Hydrolysate was filled in anaerobic storage bottles and from there it was transferred semicontinuously into various methane stages (**b**). For both, mesophilic and thermophilic acidogenesis continuous stirred tank reactors (CSTR) were used. Those were equipped with a pH sensor, which automatically regulated the inflow of NaOH for pH adjustment to 5.5 (**c**). Proteomic analysis was performed with samples from the first week of acidification (Highlighted with a *red letter* P). *Green circles* in the timeline correspond to days of taxonomic analysis (*white circles* were subjected to chemical analysis). The first column (Substr.) shows the taxonomic composition of the untreated grass biomass

Mechanically ground mixed grass (Graminidae) was acidified in three subsequent batch reactions under anaerobic conditions at mesophilic and thermophilic temperatures (Fig. 1). pH was automatically adjusted to 5.5 to prevent it dropping below that value. Acidification occurred in tap water as a result of microbial activity. The second and third batch received 5% Inoculum from the previous batch. Samples for VFA analysis were taken daily and every two days for 16S-rDNA amplicon sequencing. The mixed grass microbiome was analysed prior to entering acidification reactors, and it proved rich in Cyanobacteria- and Proteobacteria-related taxa. Upon transference into the reactors, the taxonomic profile rapidly switched to the one dominated by members of the phylum Firmicutes. This happened under both mesophilic and thermophilic conditions (Fig. 1).

After just two days, hardly any Proteobacteria and Cyanobacteria remained. As often occurs with 16S-rDNA-based analyses of plant material, cyanobacteria-related sequences may correlate to plant chloroplasts. On day four, most of the Firmicutes were suppressed by Bacteroidetes at mesophilic temperatures, while the proportion of Firmicutes remained high at 55 °C. The acidification process was repeated three times in a row and Bacteroidetes were also the dominating phylum at mesophilic temperatures. At thermophile temperatures the dominant phylum was Firmicutes, although at two of the sampling points a strong but transitory shift towards Proteobacteria was observed (Fig. 1a). In the second and third week an inoculum from the previous stages was used; however, this hardly influenced the taxonomic profile, which was constantly dominated by Bacteroidetes.

Upon termination of each acidification cycle, the high-strength liquors produced were transferred into bottles filled with nitrogen and stored at room temperature thereafter (Fig. 1b). The microbial composition in the stored liquor was analysed (Fig. 1, right) and yielded no significant changes at mesophilic temperature. However, a strong shift in the stored liquor originating from the thermophilic reaction was observed after incubation at room temperature (RT). After four days at RT, numbers of Bacteroidetes dramatically increased, yielding a stable taxonomic profile very similar to the one of the mesophilic acidification step. The microbial profile of the thermophilic samples upon RT storage was not accompanied by any changes in the concentration of chemical oxygen demand (COD) or VFA (Data not shown).

The results are in concordance with a previous work describing high titres of Bacteroidetes and Firmicutes during acidification of alginate under mesophilic conditions [24]. A microbiome dominated by Bacteroidetes and Firmicutes has also been reported for one-stage processes at mesophilic temperatures [9, 31, 32], but not for sewage sludge [7, 8].

There are no previous reports on the microbiome of acidification at thermophilic temperatures; however, a shift to Clostridia (Firmicutes) has been described for one-stage digesters [33, 34], similar to the increased titre of Firmicutes described in the present results.

### Environmental parameters

**Fig. 2:**
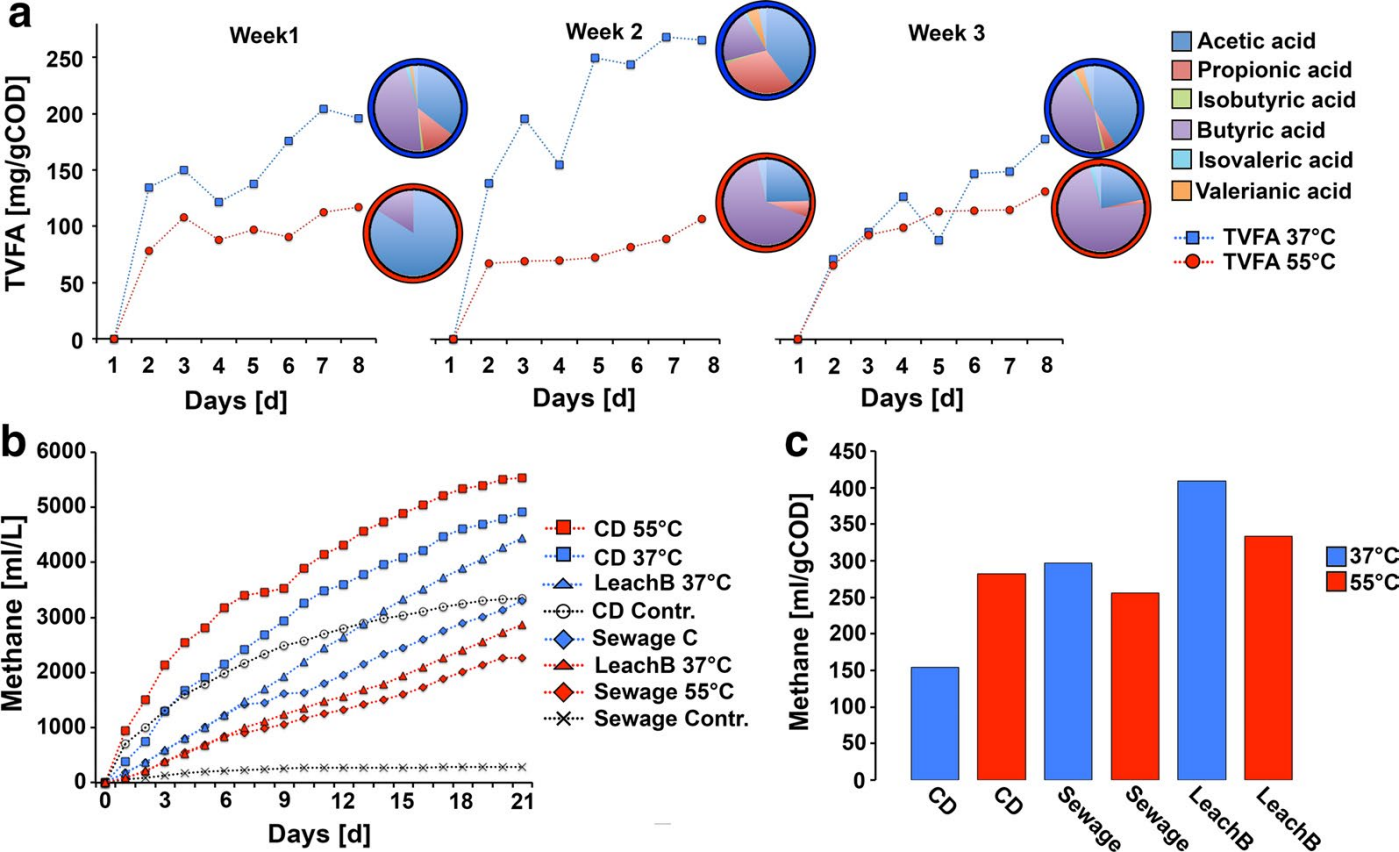
Chemical parameters during acidification and methane production: total amount of TVFA was monitored daily and samples obtained at the end of each acidification cycle were subjected to the determination of VFA spectra (**a**). Produced methane is shown as volume of methane per volume of sludge (**b**) and as volume of methane per mg of input COD (**c**)

Production of total volatile fatty acids (TVFAs) was more effective at mesophilic temperatures than at thermophilic ones (Fig. 2). With 200 mg TVFA per gram of input COD, the mesophilic stage yielded twice as many TVFAs as at thermophilic temperatures (Fig. 2a). At 37 °C, the relative amount of acetic acid and propionic acid were much higher than at 55 °C (Fig. 2b). By contrast, an accumulation of butyric acid was observed at thermophilic temperatures.

To the best of our knowledge, there are no previous reports comparing taxonomic profiles of mesophilic and thermophilic biogas acidification stages. There are reports, however, that thermophilic processes in one-stage digesters result in higher degradation efficiency compared to mesophilic ones [34–37]. Previous works have reported long incubation times for adaption of the biocoenosis to thermophile temperatures, ranging from several months [35] to up to one year [37]. Therefore, successful adaption to high temperatures and well-chosen seed sludge might be crucial for a separated acidification step.

In concordance with the reduced acid production in the thermophilic acidification, two of the corresponding methane stages (leach bed and semicontinuous batch with sewage) yielded more methane per gram of input COD with thermophilic liquor than with mesophilic. (Fig. 2c). However, in the system containing seed sludge from a co-digester (CD), the yield from the thermophilic-treated liquor was higher than in the one receiving mesophilic liquor. This might be related to the higher total solids (TS) content, high heterogeneity and high gasification activity also causing very high gas yields in the negative control from the CD sludge (Fig. 2b). In concordance with a previous work [38], the liquefied COD from the produced high-strength liquor was efficiently transformed into methane, indicating no inhibitory effects.

### Usage of the high-strength liquor produced

**Fig. 3:**
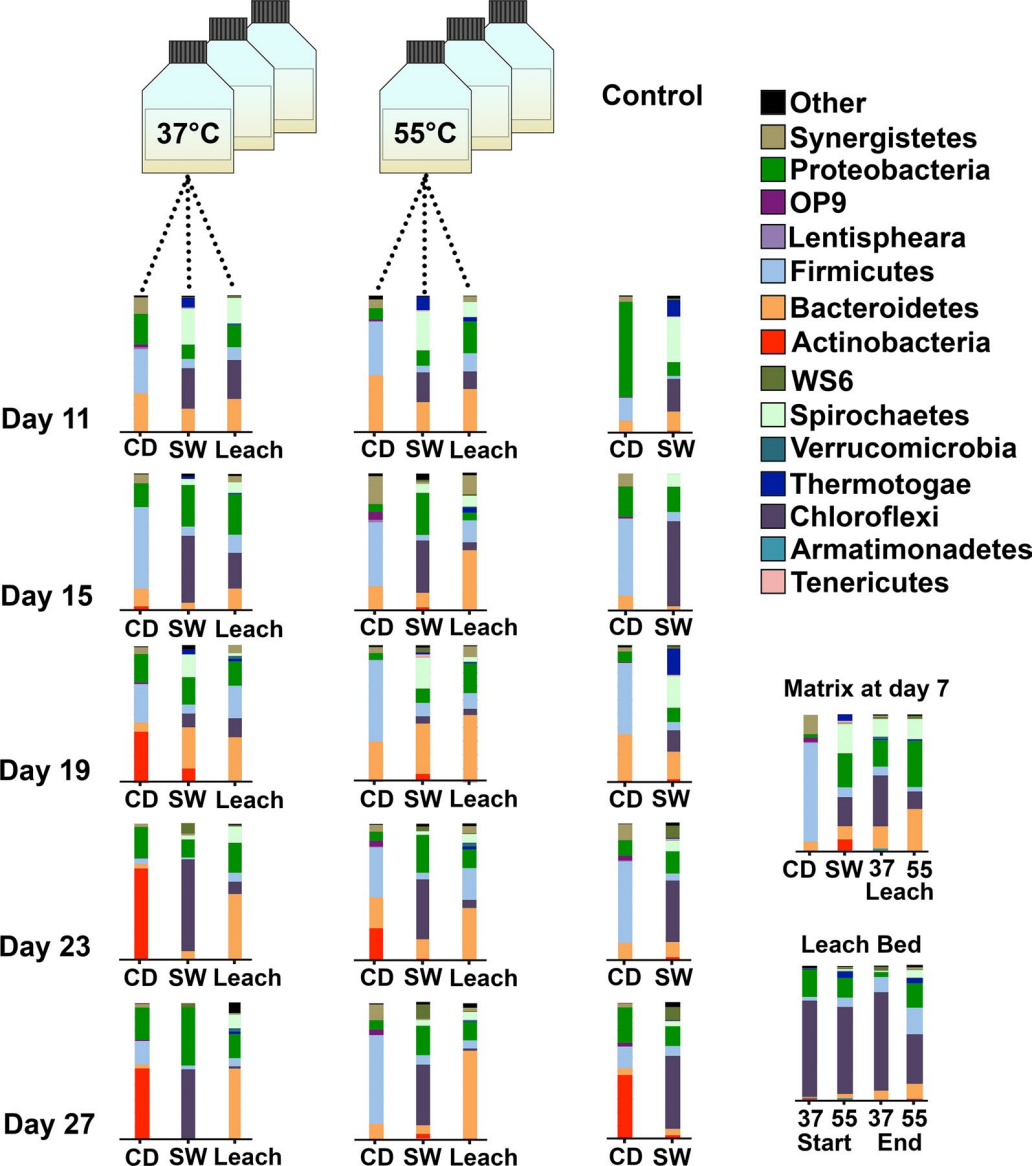
Bacterial community in the CH_4_-stages: Time-dependent taxonomic profiles at the phylum level over 20 days for various CH_4_-stages digesting hydrolysate from mesophilic and thermophilic acidification. All CH_4_-stages were performed at mesophilic temperatures. Control reactions were not fed. Taxonomic profiles of the sludges prior to the experimental setup were determined as controls, as well as the taxonomic profiles of the biofilms from the leach-bed systems. *CD* co-digester, *SW* sewage, *Leach* Leachate

High-strength liquor was stored in bottles at RT upon production, which were always flushed with nitrogen after opening to keep anoxic conditions. The liquor was semicontinuously fed into various methane stages (Figs. 1b, 2, 3).

The used seed sludge from the co-digester was very rich in Firmicutes, Synergistetes and Bacteroidetes, while the seed sludge from the sewage plant (SW) consisted mainly of Proteobacteria, Bacteroidetes, Spirochaeta and Chloroflexi (Matrix at day 7, Fig. 3). Both findings are in concordance with our previous report on several co-digester microbiomes [8]. The starting samples for the leach-bed systems (Matrix at day 7, Fig. 3) were taken 24 h after refilling the leach bed with sewage seed sludge. Compared to the original sewage, there was a dramatic decrease in Actinobacteria. This may be due to the high sensitivity of Actinobacteria to environmental changes, as sensitivity to environmental changes has been described for Actinobacteria in soil [39]. The two leach-bed systems were both rich in Chloroflexi, especially in the leach-bed biofilm (Fig. 3, Leach Bed). This is in concordance with other works describing high abundance of Chloroflexi in deep biofilm layers on building walls [40] and in the sediments of Winogradsky columns [41]. The input of the high-strength liquor, rich in Firmicutes and Bacteroidetes, did not result in an increase in those phyla in the sewage sludge batches or in the leach-bed systems (SW and Leach samples from Day 11 to Day 27, Fig. 3). Samples from both systems remained rich in Chloroflexi and Spirochaeta, even though they received a daily microbial input rich in Firmicutes and Bacteroidetes. This highlights the stability of the underlying biocoenosis and suggests the potential of separated acidification as an important step in preventing the occurrence of major microbial disturbances in the biocoenosis of the respective sewage digesters. For example, an additional thermophilic acidification stage could be included in co-digestion in sewage digesters in order to improve the robustness of the active microbiome. The positive effect of co-digesting organic matter with sewage sludge (e.g. food waste or energy grass) on the reactor performance has recently been reported [42, 43]. Moreover, the application of leachate in sewage digestion has been proposed too [44]. Our results indicate that using liquefied grass biomass (after separation from solids) might be a promising method for co-digestion with sewage. Large amounts of unused grass biomass, could still be valorised [29]. Although there have been attempts to add grass biomass into sewage sludge for co-digestion [45], co-digestion of liquefied grass biomass with sewage has not been demonstrated until now.

During the experiment, the lowered temperature in the storage bottle of the high-strength liquor at room temperature (Storage 3/Week 4, Fig. 1) resulted in a dramatically modified community composition of the thermophilic liquor after two days at RT. Thus, the transference of thermophilic high-strength liquor into a mesophilic sewage digester might destabilize the microbial community in the liquor and provide an advantage to the existing biocoenosis from the sewage digester. Using the high-strength liquor for co-digestion prevented the entry of solids into the water treatment circle.

In the last days of the experiment, the sludge samples from the co-digester exhibited low levels of Firmicutes and Bacteroidetes, and high rates of Actinobacteria. This coincided with a reduction in the production of biogas (Fig. 2). Interestingly, we have previously reported a concurrence between increasing amounts of Actinobacteria and low methane production [8]. Occurrence of Actinobacteria in anaerobic digester plants has been reported repeatedly [46, 47]. Actinobacteria were described in previous works as important key players in the degradation of plant material in compost [48] with effective enzymes that can allow large-scale application for breakdown of cellulosic plant material [49]. This is not necessarily contradictory with our results, since Actinobacteria survival and its efficiency for degradation of plant material could vary greatly at different nutrient concentrations due to their sensitivity to environmental changes as mentioned before. Although further work is needed to confirm this link, it is tempting to propose the identification and quantification of microbial key players as a marker of process efficiency.

In the case of the leach-bed system, the last part of the experiment was characterized by higher amounts of Bacteroidetes in the liquid phase (Leach samples from Day 11 to Day 27, Fig. 3). It has to be stressed that the biofilm became denser during the experiment and thus a biofilm filtering effect could be responsible for the very clear supernatant observed at the end of the process, which might, in turn, have affected the microbial composition of the leachate.

**Fig. 4:**
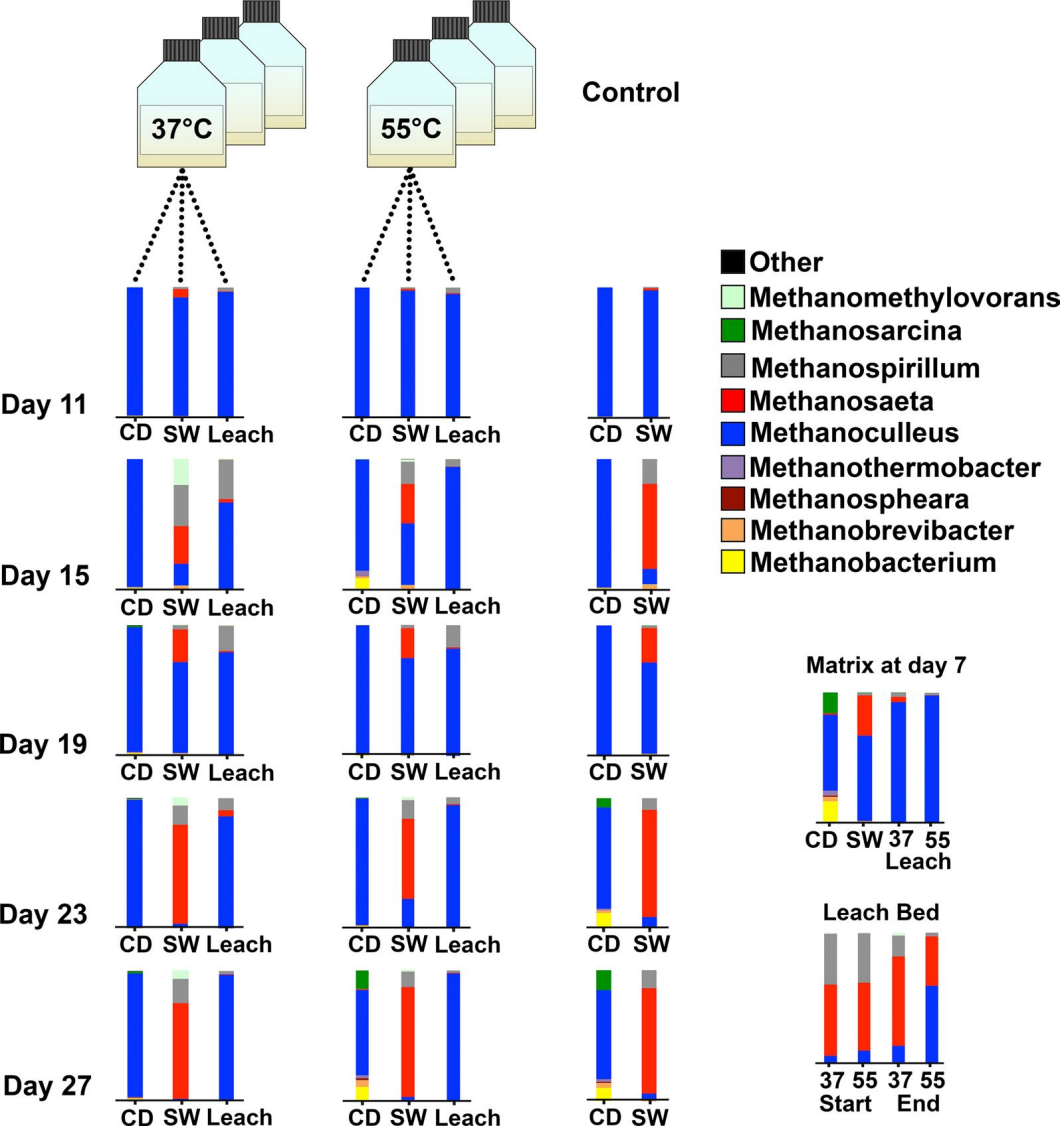
Archaeal community in the CH_4_-stages: Time-dependent community behaviour at the genus level over 20 days for various CH_4_-stages digesting hydrolysate from mesophile and thermophile acidification. All CH_4_-stage measurements were performed at mesophilic temperatures. *CD* co-digester, *SW*—sewage, *Leach* Leachate

Archaea were also detected through 16S-rDNA amplicon sequencing and identification (Fig. 4). The genus *Methanoculleus* was the most abundant one in most of the samples. The co-digester sludge contained small amounts of *Methanobacterium* and *Methanosarcina*, as previously reported for the same plant [8] (Matrix at day 7, Fig. 4). However, upon transferring the sludge into the batch systems, a rapid shift was observed, in terms of an overwhelming abundance of *Methanoculleus* (CD, SW and Leach-Bed Samples at day 11, Fig. 4). This might be related to stress factors caused by the sludge transference (e.g. changing reactor conditions or short-time exposure to oxygen), and it could be hypothesized that *Methanoculleus* is more resistant to these changes. This is consistent with previous reports on the robustness of *Methanoculleus*, which is particularly resistant to inhibitors such as ammonia [50], phenol [51] or paraffin [52].

After eight days of incubation under constant conditions *Methanosaeta* and *Methanobacterium* started to recover in the batch reactions with the sewage seed sludge (Fig. 4), although no significant increases were observed for the leach-bed system. However, *Methanosaeta* proved frequent in the biofilm from the leach bed, (Fig. 4, Leach Bed). The occurrence of *Methanosaeta* in biofilms has been reported previously [53, 54]. This result highlights the need for a separate analysis of leach-bed samples and associated biofilms. In the co-digesters, *Methanosarcina* were also recovered as of day 23 (CD samples at day 23–27, Fig. 4).

### Proteomic analysis on the high-strength liquor produced

**Fig. 5:**
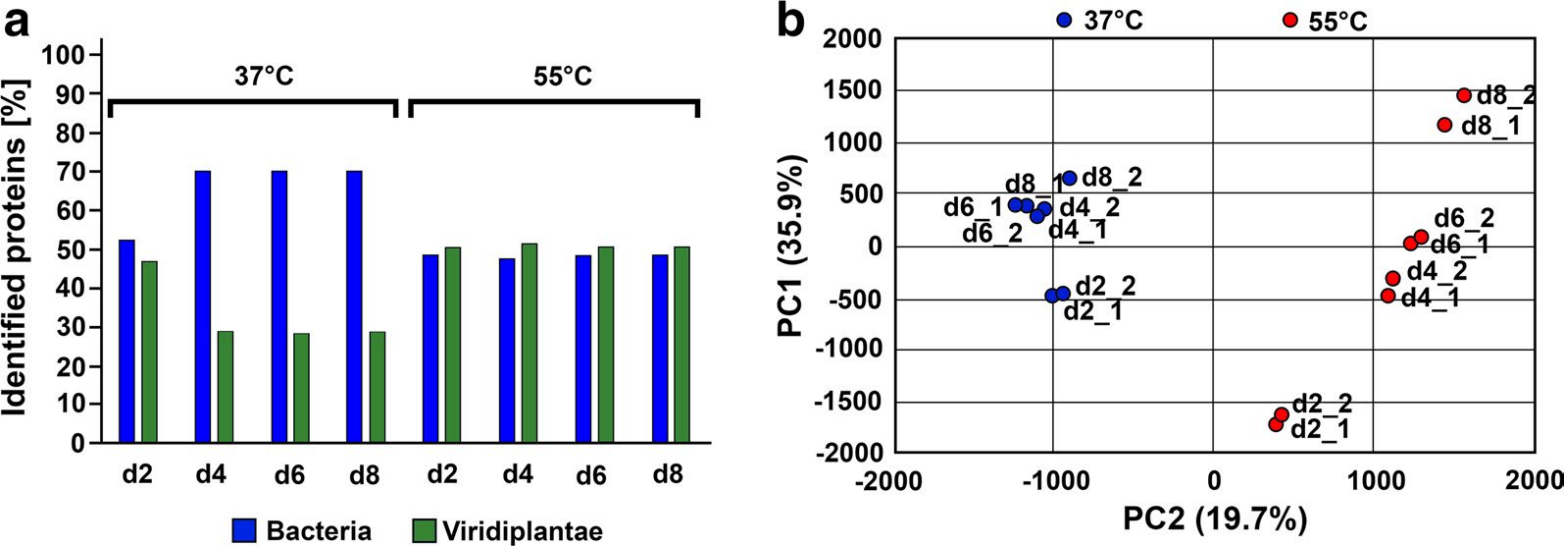
Bacteria and Viridiplantae proteomic profile evolution in the first cycle of acidification (**a**); PCA aggrupation of quantified peptides at mass spectroscopy analysis (**b**)

Proteins were extracted from the samples d2, d4, d6 and d8 from the first cycle of acidification. The proteome at mesophilic and thermophilic temperatures proved strikingly different in the previous SDS-PAGE analysis (Additional file 1: Figure S3). This observation was approved by a principal component aggrupation (PCA) from mass spectroscopy raw data (peptide) analysis, where samples not only separated into two groups by temperature (*X*-axis, Fig. 5b), but also showed dynamic changes in time (*Y*-axis, Fig. 5b).

At the first stages, plant proteins were detected in the greatest amounts, as expected from the mixed grass biomass used in all assays. However, in line with increasing incubation time, the ratio between plants and bacteria shifted due to massive microbial growth and/or degradation of plant material at 37 °C (Fig. 5a). However at 55 °C, there was a constant plant:bacteria ratio in the protein abundance, indicating a decrease in the total microbial population.

An abundance of enzymes involved in carbohydrate metabolism and degradation in metaproteomes from both series of samples were identified using a protein database search for Bacteria and Archaea domains, although additionally diverse chaperones and heat-shock proteins (e.g. 10 and 60 kDa chaperonins, and GroEL) were overrepresented in the thermophilic samples (Additional file 2: Table S4). Among the most abundant proteins detected in all analysed samples, there was an almost complete set of glycolytic enzymes (glucose-6-phosphate isomerase, fructose-bisphosphate aldolase, triosephosphate isomerase, glyceraldehyde-3-phosphate dehydrogenase, phosphoglycerate kinase, enolase), as well as components of sugar transport systems (like the phosphotransferase system, PTS). These results indicate that the microbial population is basically engaged in the degradation and catabolism of sugars in the fermentative phase of short-chain acid production, an observation that is coherent with previous reports on the metaproteome [28] and metametabolome [55] of this kind of microbial communities.

**Fig. 6:**
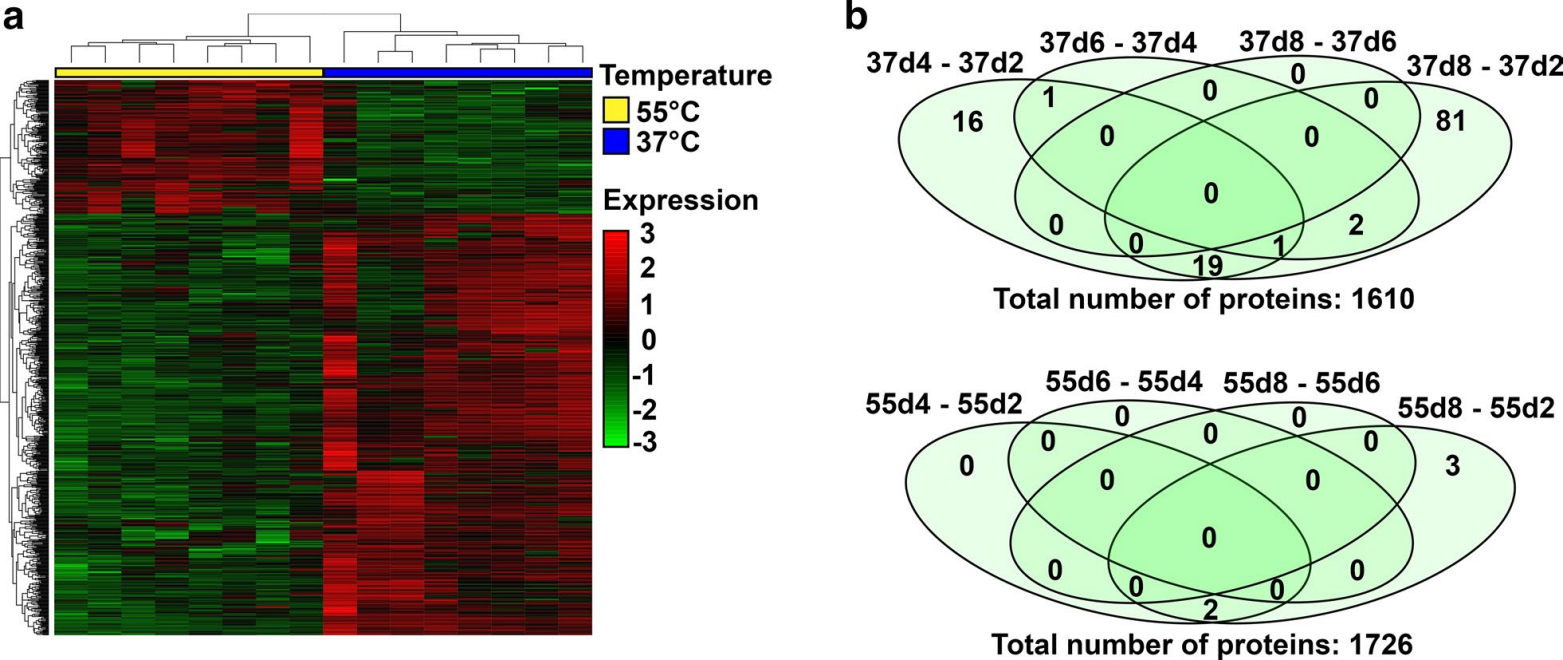
Proteomic differences between 37 and 55 °C: HCT for differentially expressed proteins between mesophilic and thermophilic conditions (**a**). Number of differentially expressed proteins (*p* value < 0.05) over time at two different culture temperatures: 37 °C (*upper Venn diagram*) and 55 °C (*lower Venn diagram*) (**b**)

Label-free quantitative proteome analysis was performed to determine differentially expressed proteins between mesophilic and thermophilic temperatures (Additional file 3: Table S5). A total of 1731 proteins were quantified from samples d2, d4, d6 and d8 collected from the first cycle of acidification: 556 proteins increased and 176 decreased between mesophilic and thermophilic conditions (37 vs. 55 °C). Samples were compared using the Limma statistics software package. Differences in protein abundances clearly separated samples into two clusters corresponding to culture temperature, with the subset of proteins showing an increased expression that was richer at 37 than 55 °C (Fig. 6a). On comparing protein abundances during sampling time, 120 (out of 1731) proteins showed differential expression at 37 °C, whereas at 55 °C, the differentially expressed proteins were only 5 (out of 1731) (Fig. 6b). Remarkably, most differences were observed when comparing d2 and d4 samples, and d2 and d8 at mesophilic conditions, whereas at thermophilic conditions a small set of differential proteins was only detected when comparing samples d2 and d8 (Fig. 6b). Among the differentially expressed proteins at mesophilic conditions there is a notable representation of ribosomal proteins indicating a dynamic state of these microbial communities. The taxonomic profiles of metaproteome samples were in agreement with the presented metagenomic data.

Among the differentially expressed proteins in d2 samples at 37 °C, noteworthy was the presence of several membrane transport systems from Firmicutes species involved in sugar uptake. These were the PTS HPr-related protein and the cellobiose-specific PTS IIB component, and the PTS phosphocarrier protein HP. There was also an increase in haemolysin-type calcium-binding protein, with a predicted hydrolytic activity on *O*-glycosyl compounds and a carbohydrate-binding domain (CBM) type 2 from an Alpha-proteobacterium in d2 samples. Previous studies on mesophilic biogas-producing, cellulolytic communities have indicated the abundance of sugar transporters and enzymes involved in polysaccharide degradation [9, 28, 56].

## Conclusions

Plant biomass (a mix of grass) was acidified at mesophilic and thermophilic temperatures. The taxonomic communities in both cases proved very different, and consisted of Bacteroidetes and Firmicutes at 37 °C and Firmicutes and Proteobacteria at 55 °C. At the methane stage, *Methanosaeta*, *Methanomicrobium* and *Methanosarcina* proved highly sensitive to environmental changes whereas *Methanoculleus* proved to be very robust with all the seed sludges. At the end of the experiment, there was an increase in Actinobacteria in the semicontinuous batches containing co-digester seed sludge, which coincided with reduced biogas formation. Thus, Actinobacteria determination could be a useful prediction tool for biogas production.

Metaproteome analyses only detected significant expression differences in mesophilic samples, and collectively implied a dynamic microbial community engaged in polysaccharide demolition and sugar fermentation as remarkable metabolic activities during the acidification phase. Thermophilic samples showed more stable protein composition with an abundance of chaperones suggesting a role in protein stability under thermal stress.

## Methods

### Reactor performance

Acidification of grass was carried out in three sequential reactions, which were operated in parallel at 37 and 55 °C with a COD input concentration of 30 gO_2_/L. Acidification occurred in tap water as a result of microbial activity. For the second and third cycle of acidification 5% inoculum was applied from the previous reactions. After separating the liquid phase from the solids manually using a sieve, the resulting high-strength liquor was stored under anaerobic conditions (nitrogen atmosphere) until further fermentation occurred in several methane stages (Fig. 1). Acidification was carried out in continuous stirred tank reactors with a total working volume of 5 L and equipped with a pH regulation system (BL 7916, Hanna Instruments, Germany) that stabilized the pH at 5.5 (Fig. 1).

The high-strength liquor was stored until usage in glass bottles at RT. To ensure anaerobic atmosphere, the bottles were nitrogen-purged and a gasbag (TECOBAG, TESSERAUX Spezialverpackungen GmbH, Germany) was connected to verify that no further gas production occurred.

High-strength liquor was digested in semicontinuous batch reactions, as well as two leach-bed systems. The setup of batch systems was carried out according to VDI 4630 [57]. Feeding was applied not only at the beginning of the experiment but semicontinuously by adding daily 33 mL/L day to the batch bottles, which corresponds to a solubilized COD of 0.51 gO2 for the mesophilic stage and 0.39 gO2/L for the thermophilic stage.

The leach-bed systems consisted of packed columns with 3 L working volumes. They were filled with 2 L of seed sludge and 485 g of bed packing (Hel-X-Füllkörper, Christian Stöhr GmbH&Co.KG, Germany) and were fed equally to the batch bottles with 33 mL/L*day. Gas production was quantified with a MilliGascounter (Ritter Apparatebau GmbH, Germany) and collected in a gasbag for further analysis (TECOBAG, TESSERAUX Spezialverpackungen GmbH, Germany).

In total, eight methane stages were set in place. Two leach-bed systems, three batch systems filled with low TS seed sludge (sewage) and three batch systems filled high TS seed sludge (CSTR, co-digester) (Additional file 4: Table S1 and Additional file 5: Table S2). The leach-bed systems were filled with sewage sludge and the leach bed contained a thick biofilm from previous experiments also performed with sewage. All methane stages were set as duplets in order to compare methanisation of liquor from acidification, at 37 and 55 °C. Control reactions without feeding were performed (Fig. 1).

### Sampling and environmental chemical analysis

A mixture of grass species was collected from a backyard in Jena (Germany) and mechanically ground. Mechanical treatment was performed using a conventional juicer (Angel Juicer 8500 s, Angel Co.LTD., Corea). After the mechanical treatment, grass juice and squashed solids were remixed and stored at −20 °C until use.

Sewage was collected from a water treatment plant in Jena (Jena). Sludge from a co-digester was collected from the continuous stirred tank reactor near the water treatment plant. Sludge samples and substrates were characterized by analysing TS and VS (Additional file 4: Table S1) and during the acid-producing step, the concentration of VFA and COD was monitored daily using conventional photometer-based assays (Nanocolor CSB15000 and Nanocolor organische Säuren 3000, Macherey-Nagel, Germany) (Fig. 2). At the end of each experiment, the VFA spectrum was determined at Eurofins Umwelt Ost GmbH, using a gas chromatograph (Shimadzu, Japan). A flame ionization detector was equipped with a DB.1701 column (Macherey-Nagel, Germany).

During methanisation of the high-strength liquor produced, the volume of biogas obtained was monitored daily, using a “COMBIMASS GA-m” gas measurement device (Binder, Germany), to determine the ratio of CO_2_ and CH_4_ (Fig. 2). Samples for DNA analysis were taken every two days for the acidification step and every four days for the methane stages. One milliliter of sample was mixed with 1 mL of pure ethanol and kept at −20 °C until required. Additional samples from the acidification stages were taken for proteomic approaches (20 mL per sample). Samples for proteomic analysis were stored at −70 °C until further analysis.

### DNA extraction and amplification

To reduce the amount of humic acids and other inhibitors, samples were intensively washed: they were centrifuged at 20,000×*g* and resuspended in PBS buffer repeatedly until a clear supernatant was observed. DNA Extraction was performed using the PowerSoil DNA isolation KIT (Mo Bio Laboratories, USA). After a quality control on a 0.8% (w/v) agarose gel and quantification with the Nanodrop-1000 Spectrophotometer (Thermo Scientific, Wilmington, DE, USA), variable regions V1–V3 from the 16S-rDNA gene were amplified. For amplification of bacterial 16S-rDNA sequences the universal primers 28F (5′-GAG TTT GAT CNT GGC TCA G-3′) and 519R (5′-GTN TTA CNG CGG CKG CTG-3′) were used (Additional file 6: Table S6 and Additional file 7: Table S7). Archaea target sequences were amplified using the primers Arch349F (5′-GYG CAS CAG KCG MGA AW-3′) and Ar9r (5′-CCC GCC AAT TCC TTT AAG TTTC-3′) (Additional file 8: Table S8). Resulting amplicons had a length of 500 bp for bacteria and 578 bp for archaea [58]. For amplification, after initial denaturation at 95 °C for 5 min, 35 cycles of amplification (95 °C for 30 s, 54 °C for 30 s, and extension at 72 °C for 1 min) were carried out. The reaction was completed with a 10-min elongation step at 72 °C.

### DNA-sequencing and analysis

All DNA-sampled were quantified using the Qubit^®^ 2.0 Fluorometer (Invitrogen, Carlsbad, CA, USA). For bacteria and archaea, separate libraries were built. Approximately 100 ng of each sample was added applying the amplicon fusion method (Ion Plus Fragment Library Kit, MAN0006846, Life Technologies). For quantification, the Agilent 2100 Bioanalyzer (Agilent Technologies Inc, Palo Alto, CA, USA) was used. PCRs were carried out applying the Ion PGM Template OT2 400 kit as stated by the manufacturer (MAN0007218, Revision 3.0 Life Technologies). For the final sequencing step, an Ion 318 Chip v2 on a Personal Genome Machine (PGM) (IonTorrentTM, Life Technologies) at Life Sequencing S.L. (Life Sequencing, Valencia, Spain) was used. Here the Ion PGM Sequencing 400 kit was applied, following the manufacturer’s instructions (publication number MAN0007242, revision 2.0, Life Technologies).

After removing short (<100 bp) and low-quality (<q15) reads, resulting sequences were split and barcode sequences were trimmed. Final sequences were then analysed using Mothur [59]. Based on the k-mer algorithm, sequences were aligned to the 16S reference from the Greengenes database. In the case of eubacteria, assignments were performed at the phylum level. Assignments with a similarity percentage lower than 70% were not considered for further analysis. In case of archaea, amplicons were analysed at the genus level and the cut-off was set to 93%.

### Protein extraction, identification and data analysis

Protein extraction was performed using the NoviPure Soil Protein Extraction Kit (MO BIOS Laboratories Inc). Total protein extracts were precipitated with TCA (Trichloroacetic Acid) to clean total extracts, and pellets were dissolved with 100 µL of 8 M Urea, 0.5 M TEAB (Triethylammonium bicarbonate buffer). The protein concentration in the samples was determined using Qubit^®^ 2.0 Fluorometer (Invitrogen, Carlsbad, CA, USA). Then, 20 µg of each sample was digested as described in the following protocol. Cysteine residues were reduced by 2 mM DTT (DL-Dithiothreitol) in 50 mM ABC (Ammonium bicarbonate) at 60 °C for 20 min. Sulphydryl groups were alkylated with 5 mM IAM (iodoacetamide) in 50 mM ABC in the dark at room temperature for 30 min. IAM excess was neutralized with 10 mM DTT in 50 mM ABC, 30 min at room temperature. Each sample was subjected to trypsin digestion with 500 ng (100 ng/µL) of sequencing grade-modified trypsin (Promega) in 50 mM ABC at 37 °C overnight. The reaction was stopped with TFA (trifluoroacetic acid) at a final concentration of 0.1%. Final peptide mixture was concentrated in a speed vacuum and suspended in 65 µL of 2% CAN, 0.1%TFA. Finally, 1.5 µg of each sample was used for protein identification by LC_MS/MS analysis and label-free differential expression analysis. For that 5 µL of each sample was loaded onto a trap column (NanoLC Column, 3 µm C18-CL, 75 µm × 15 cm; Eksigent) and desalted with 0.1% TFA at 3 µL/min during 10 min. The peptides were then loaded onto an analytical column (LC Column, 3 µ C18-CL, 75 µm × 12 cm, Nikkyo) equilibrated in 5% acetonitrile 0.1% FA (formic acid). Elution was carried out with a linear gradient from 5a35% B in A for 120 min. (A: 0.1% FA; B: ACN, 0.1% FA) at a flow rate of 300 nL/min. Peptides were analysed in a mass spectrometer nanoESI-qQTOF (5600 TripleTOF, ABSCIEX).

Eluted peptides were ionized applying 2.8 kV to the spray emitter. Analysis was carried out in a data-dependent mode. Survey MS1 scans were acquired from 350–1250 m/z for 250 ms. The quadrupole resolution was set to ‘UNIT’ for MS2 experiments, which were acquired 100–1500 m/z for 50 ms in ‘high sensitivity’ mode. Following which switch criteria were used: charge: 2+ to 5+; minimum intensity; 70 counts per second (cps). Up to 25 ions were selected for fragmentation after each survey scan. Dynamic exclusion was set to 25 s.

ProteinPilot default parameters were used to generate peak list directly from 5600 TripleTof wiff files. The Paragon algorithm of ProteinPilot v 4.5 was used to search Uniprot bacteria and Archaea protein database with the following parameters: trypsin specificity, cys-alkylation and the search effort set to through with FDR to multiple test correction.

To avoid using the same spectral evidence in more than one protein, the identified proteins were grouped based on MS/MS spectra by the ProteinPilot Pro group algorithm. The Peak View v1.1 (SCIEX) software was used to generate peptide and protein areas from ProteinPilot result files and to perform a multivariant data analysis.

Differential expression analysis was performed using the Limma package (http://bioconductor.org/packages/limma/), fitting a linear model using an appropriate design matrix. A contrast matrix was set to make comparisons of interest, in our case 37 versus 55 °C. For the contrast of interest the package computed fold changes and t-statistics. After fitting a linear model, the standard errors were moderated using an empirical Bayes method to obtain moderated t-statistics. The function top Table was used to present a list of the proteins most likely to be differentially expressed for a given contrast. FDR was used to adjust the *p* value for multiple testing.

## Additional files

**Additional file 1: Figure S3.** SDS-PAGE displaying the protein profiles.

**Additional file 2: Table S4.** Mascot results.

**Additional file 3: Table S5.** Differentially expressed proteins.

**Additional file 4: Table S1.** Description of the used seed sludge.

**Additional file 5: Table S2.** Overview of reaction stages and reactor performance.

**Additional file 6: Table S6.** Number of sequences and mean length for bacteria from the acidification stages.

**Additional file 7: Table S7.** Number of reads and mean length of reads for bacteria from the methane stages.

**Additional file 8: Table S8.** Number of reads and mean length of reads for archaea from the methane stages.

## Abbreviations

CD: co-digester (sludge from industrial CSTR)
COD: chemical oxygen demand
CSTR: continuous stirred tank reactor
LB: leach bed
Leach: Leachate from leach-bed system
RT: room temperature
SW: sewage
TS: total solids
TVFA: total volatile fatty acids
VFA: volatile fatty acids
