# Supplementary material for: From grass to gas: microbiome dynamics of grass biomass acidification under mesophilic and thermophilic temperatures
Source: Biotechnol Biofuels. 2017 Jul 3;10:171. doi: 10.1186/s13068-017-0859-0 (PMC5496412; doi:10.1186/s13068-017-0859-0)

Fig. S3: SDS-page: Blue-Coomassie stained gel showing protein extraction from several samples. Molecular weights are given to the left in kDa (10 µg per lane).
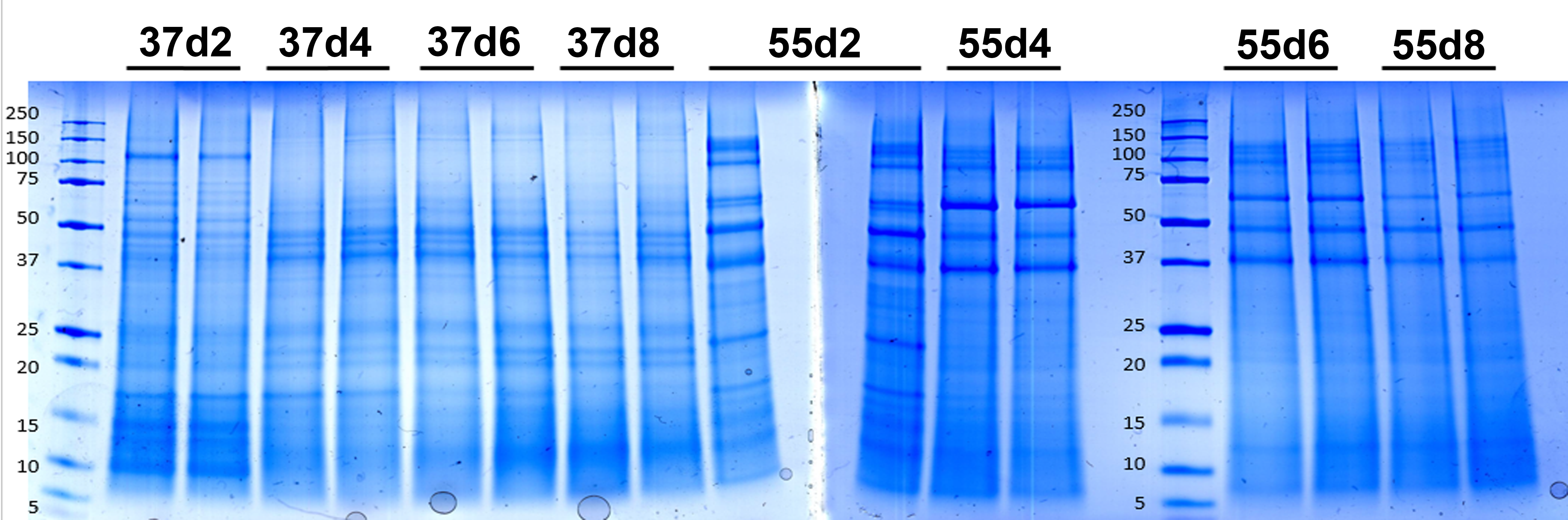

Supplement: Supplementary file 1 — Additional file 1: Figure S3. SDS-PAGE displaying the protein profiles. [file 13068_2017_859_MOESM1_ESM.docx]
