# Supplementary material for: From grass to gas: microbiome dynamics of grass biomass acidification under mesophilic and thermophilic temperatures
Source: Biotechnol Biofuels. 2017 Jul 3;10:171. doi: 10.1186/s13068-017-0859-0 (PMC5496412; doi:10.1186/s13068-017-0859-0)
Supplement: Supplementary file 4 — Additional file 4: Table S1. Description of the used seed sludge. [file 13068_2017_859_MOESM4_ESM.docx]

Tab. S1: Description of the used seed sludge

| **Stage of the experiment** | **Type of seed sludge** | **Origin of seed sludge** | **Input at the original plant** | **Plant configuration** |
| --- | --- | --- | --- | --- |
| Digestion of high strength liquor in high-TS sludge | High-TS sludge from a plug flow digester  (17% TS) | Two-stage digester from Jena (vertical plug flow and CSTR) | Farm manure, Livestock  Farming waste, Silage | HRT: 87 days  Produced gas: 1.2 m3/m3 × day  OLR: 3.0 kg × VS/m3 × day  Stage 1 (plug flow): 790 m3  Stage 2 (CSTR): 2000 m3  Stage 3 (Digestate): 3800 m3 |
| Digestion of high strength liquor in low-TS sludge | Low-TS sludge from a sewage digester (4% TS) | Digester from the sewage plant in Jena (CSTR) | Municipal sewage sludge | HRT: 21 days  Produced gas: 0.6 m3/m3 × day  OLR: 1.8 kg × VS/m3 × day Single stage process (2 reactors)  Digester volume: 2 × 2000 m3 |
| Digestion of high strength liquor in a leach-bed system | Low-TS sludge from a sewage digester (4% TS)  Fully overgrown solids for a packed bed | Digester from the sewage plant in Jena (CSTR) | Municipal sewage sludge | HRT: 21 days  Produced gas: 0.6 m3/m3 × day  OLR: 1.8 kg × VS/m3 × day Single stage process (2 reactors)  Digester volume: 2 × 2000 m3 |
