# Supplementary material for: From grass to gas: microbiome dynamics of grass biomass acidification under mesophilic and thermophilic temperatures
Source: Biotechnol Biofuels. 2017 Jul 3;10:171. doi: 10.1186/s13068-017-0859-0 (PMC5496412; doi:10.1186/s13068-017-0859-0)
Supplement: Supplementary file 5 — Additional file 5: Table S2. Overview of reaction stages and reactor performance. [file 13068_2017_859_MOESM5_ESM.docx]

Tab. S2: Overview of reaction stages and reactor performance

|  | **Experiment 1** | **Experiment 2** |
| --- | --- | --- |
| **Acidification stages** | Temperature = 37°C;  pH 5.5 (NaOH-regulated);  CSTR (Batch);  Incubation per cycle = 7day;  Volume = 5L;  Input = 30 gO_2_/L;  No seed sludge;  Duration: 3 × 7days;  5% inoculum (from previous batch cycle) | Temperature = 55°C;  pH 5.5 (NaOH-regulated);  CSTR (Batch);  Incubation per cycle = 7day;  Volume = 5L;  Input = 30 gO_2_/L;  No seed sludge;  Duration: 3 × 7days;  5% inoculum (from previous batch cycle) |
|  |  |  |
|  |  |  |
|  |  |  |
|  |  |  |
| **Methane stage 1** | Seed sludge: Industrial codigester (CSTR);  Temperature = 37°C;  Substrate = Mesophilic liquor;  Input: 33 ml/day (0.51 gO_2_/L×day);  Semi-continuous Batch;  1L bottles (horizontal shaking)  No pH regulation | Seed sludge: Industrial codigester (CSTR);  Temperature = 37°C;  Substrate = Thermophilic liquor;  Input: 33 ml/day (0.39 gO_2_/L×day);  Semi-continuous Batch;  1L bottles (horizontal shaking);  No pH regulation |
| **Methane stage 2** | Seed sludge: Sewage sludge  Temperature = 37°C;  Substrate = Mesophilic liquor;  Input: 33 ml/day (0.51 gO_2_/L×day);  Semi-continuous Batch;  1L bottles (horizontal shaking)  No pH regulation | Seed sludge: Sewage sludge  Temperature = 37°C;  Substrate = Mesophilic liquor;  Input: 33 ml/day (0.37 gO_2_/L×day);  Semi-continuous Batch;  1L bottles (horizontal shaking)  No pH regulation |
| **Methane stage 3** | Seed sludge: Sewage sludge  Temperature = 37°C;  Substrate = Mesophilic liquor;  Input: 33 ml/day (0.51 gO_2_/L×day);  Semi-continuous Batch;  Volume = 3L  Leach bed (Hel-X-Füllkörper);  Continuous leach circulation;  No pH regulation | Seed sludge: Sewage sludge  Temperature = 37°C;  Substrate = Mesophilic liquor;  Input: 33 ml/day (0.37 gO_2_/L×day);  Semi-continuous Batch;  Volume = 3L  Leach bed (Hel-X-Füllkörper);  Continuous leach circulation;  No pH regulation |
