# Supplementary material for: From grass to gas: microbiome dynamics of grass biomass acidification under mesophilic and thermophilic temperatures
Source: Biotechnol Biofuels. 2017 Jul 3;10:171. doi: 10.1186/s13068-017-0859-0 (PMC5496412; doi:10.1186/s13068-017-0859-0)
Supplement: Supplementary file 6 — Additional file 6: Table S6. Number of sequences and mean length for bacteria from the acidification stages. [file 13068_2017_859_MOESM6_ESM.docx]

Tab. S6: Number of sequences and mean length for bacterial reads from the acidification stages

| **Name of sample** | **Reads** | **Mean length** |
| --- | --- | --- |
| Grass - Substrate | 9,195 | 410 |
| Acidification W1-37-d2 | 1,871 | 305 |
| Acidification W1-55-d2 | 3,566 | 369 |
| Acidification W1-37-d4 | 8,565 | 350 |
| Acidification W1-55-d4 | 2,820 | 323 |
| Acidification W1-37-d6 | 35,180 | 368 |
| Acidification W1-37-d8 | 842 | 377 |
| Acidification W1-55-d8 | 2,124 | 298 |
| Acidification W2-37-d2 | 1,390 | 327 |
| Acidification W2-55-d2 | 3,901 | 327 |
| Acidification W2-37-d4 | 1,101 | 370 |
| Acidification W2-55-d4 | 788 | 453 |
| Acidification W2-37-d6 | 3,058 | 392 |
| Acidification W2-55-d6 | 1,265 | 395 |
| Acidification W2-37-d8 | 595 | 395 |
| Acidification W2-55-d8 | 1,824 | 377 |
| Acidification W3-37-d2 | 4,088 | 383 |
| Acidification W3-55-d2 | 5,309 | 376 |
| Acidification W3-37-d4 | 1,086 | 336 |
| Acidification W3-55-d4 | 4,476 | 339 |
| Acidification W3-37-d6 | 3,044 | 398 |
| Acidification W3-55-d6 | 1,917 | 447 |
| Acidification W3-37-d8 | 253,369 | 308 |
| Acidification W3-55-d8 | 1,018 | 427 |
| Acidification W3-RT37-d2 | 1,660 | 415 |
| Acidification W3-RT55-d2 | 1,439 | 376 |
| Acidification W3-RT37-d4 | 788 | 373 |
| Acidification W3-RT55-d4 | 24,697 | 446 |
| Acidification W3-RT37-d6 | 1,359 | 389 |
| Acidification W3-RT55-d6 | 1,772 | 388 |
| Acidification W3-RT37-d8 | 577 | 383 |
| Acidification W3-RT55-d8 | 1,043 | 413 |
