# Supplementary material for: From grass to gas: microbiome dynamics of grass biomass acidification under mesophilic and thermophilic temperatures
Source: Biotechnol Biofuels. 2017 Jul 3;10:171. doi: 10.1186/s13068-017-0859-0 (PMC5496412; doi:10.1186/s13068-017-0859-0)
Supplement: Supplementary file 7 — Additional file 7: Table S7. Number of reads and mean length of reads for bacteria from the methane stages. [file 13068_2017_859_MOESM7_ESM.docx]

Tab. S7: Number of reads and mean length of reads for bacteria from the methane stages

| **Name of sample** | **Reads** | **Mean length** |
| --- | --- | --- |
| C-Euco-d0 | 864 | 365 |
| 37-Euco-d4 | 1,768 | 408 |
| 55-Euco-d4 | 1,405 | 398 |
| C-Euco-d4 | 521 | 410 |
| 37-Euco-d8 | 883 | 401 |
| 55-Euco-d8 | 4,545 | 341 |
| C-Euco-d8 | 764 | 391 |
| 37-Euco-d12 | 1,842 | 390 |
| 55-Euco-d12 | 1,485 | 409 |
| C-Euco-d12 | 981 | 402 |
| 37-Euco-d16 | 1,155 | 379 |
| 55-Euco-d16 | 1,089 | 412 |
| C-Euco-d16 | 1,614 | 377 |
| 37-Euco-d20 | 2,263 | 380 |
| 55-Euco-d20 | 2,083 | 371 |
| C-Euco-d20 | 2,825 | 371 |
| C-SW-d0 | 602 | 421 |
| 37-SW-d4 | 2,624 | 422 |
| 55-SW-d4 | 2,910 | 431 |
| C-SW-d4 | 1,931 | 418 |
| 37-SW-d8 | 1,636 | 424 |
| 55-SW-d8 | 1,732 | 420 |
| C-SW-d8 | 577 | 420 |
| 37-SW-d12 | 1,776 | 376 |
| 55-SW-d12 | 33,005 | 373 |
| C-SW-d12 | 822 | 433 |
| 37-SW-d16 | 444 | 405 |
| 55-SW-d16 | 1,079 | 410 |
| C-SW-d16 | 64,878 | 354 |
| 37-SW-d20 | 2,982 | 309 |
| 55-SW-d20 | 23,503 | 326 |
| C-SW-d20 | 17,184 | 358 |
| Biofilm-Start-37 | 1,403 | 383 |
| Biofilm-Start-55 | 2,566 | 405 |
| Biofilm-End-37 | 710 | 400 |
| Biofilm-End-55 | 16,395 | 409 |
| Leach-37-d0 | 959 | 396 |
| Leach-55-d0 | 724 | 396 |
| Leach-37-d4 | 2,259 | 409 |
| Leach-55-d4 | 1,797 | 383 |
| Leach-37-d8 | 1,338 | 405 |
| Leach-55-d8 | 4,205 | 417 |
| Leach-37-d12 | 503 | 414 |
| Leach-55-d12 | 19,397 | 453 |
| Leach-37-d16 | 947 | 413 |
| Leach-55-d16 | 12,437 | 414 |
| Leach-37-d20 | 55,342 | 356 |
| Leach-55-d20 | 1,268 | 396 |
| L1A-55-d6 | 2,594 | 416 |
