# Supplementary material for: From grass to gas: microbiome dynamics of grass biomass acidification under mesophilic and thermophilic temperatures
Source: Biotechnol Biofuels. 2017 Jul 3;10:171. doi: 10.1186/s13068-017-0859-0 (PMC5496412; doi:10.1186/s13068-017-0859-0)
Supplement: Supplementary file 8 — Additional file 8: Table S8. Number of reads and mean length of reads for archaea from the methane stages. [file 13068_2017_859_MOESM8_ESM.docx]

Tab. S8: Number of reads and mean length of reads for archaea from the methane stages

| **Name of sample** | **Reads** | **Mean length** |
| --- | --- | --- |
| C-Euco-d0 | 4,121 | 173 bp |
| 37-Euco-d4 | 16,665 | 396 bp |
| 55-Euco-d4 | 9,291 | 341 bp |
| C-Euco-d4 | 8,886 | 260 bp |
| 37-Euco-d8 | 11,629 | 226 bp |
| 55-Euco-d8 | 4,106 | 315 bp |
| C-Euco-d8 | 11,771 | 131 bp |
| 37-Euco-d12 | 11,322 | 302 bp |
| 55-Euco-d12 | 10,613 | 409 bp |
| C-Euco-d12 | 10,138 | 380 bp |
| 37-Euco-d16 | 35,098 | 228 bp |
| 55-Euco-d16 | 15,843 | 383 bp |
| C-Euco-d16 | 1,891 | 207 bp |
| 37-Euco-d20 | 27,677 | 301 bp |
| 55-Euco-d20 | 1,511 | 225 bp |
| C-Euco-d20 | 15,812 | 227 bp |
| C-SW-d0 | 20,431 | 255 bp |
| 37-SW-d4 | 51,179 | 218 bp |
| 55-SW-d4 | 19,73 | 395 bp |
| C-SW-d4 | 17,777 | 300 bp |
| 37-SW-d8 | 26,646 | 418 bp |
| 55-SW-d8 | 21,654 | 274 bp |
| C-SW-d8 | 14,833 | 319 bp |
| 37-SW-d12 | 27,206 | 418 bp |
| 55-SW-d12 | 15,998 | 368 bp |
| C-SW-d12 | 25,793 | 377 bp |
| 37-SW-d16 | 11,178 | 294 bp |
| 55-SW-d16 | 53,94 | 159 bp |
| C-SW-d16 | 40,778 | 200 bp |
| 37-SW-d20 | 39,236 | 291 bp |
| 55-SW-d20 | 58,3 | 331 bp |
| C-SW-d20 | 27,957 | 422 bp |
| Biofilm-Start-37 | 17,929 | 400 bp |
| Biofilm-Start-55 | 10,851 | 444 bp |
| Biofilm-End-37 | 9,000 | 456 bp |
| Biofilm-End-55 | 30,565 | 292 bp |
| Leach-37-d0 | 19,021 | 283 bp |
| Leach-55-d0 | 17,221 | 283 bp |
| Leach-37-d4 | 10,17 | 372 bp |
| Leach-55-d4 | 10,688 | 275 bp |
| Leach-37-d8 | 27,395 | 346 bp |
| Leach-55-d8 | 40,587 | 271 bp |
| Leach-37-d12 | 12,174 | 310 bp |
| Leach-55-d12 | 23,277 | 237 bp |
| Leach-37-d16 | 198 | 379 bp |
| Leach-55-d16 | 16,404 | 374 bp |
| Leach-37-d20 | 15,678 | 407 bp |
| Leach-55-d20 | 20,677 | 318 bp |
